# Sulfur-Containing Heterocyclic Aromatic Hydrocarbons Alter Estrogen Metabolism and Cause DNA Damage and Apoptosis in Granulosa Cells

**DOI:** 10.3390/ijms26168004

**Published:** 2025-08-19

**Authors:** Genevieve A. Perono, Thane Tomy, Kara Loudon, Laiba Jamshed, Bianca Garlisi, Sylvia Lauks, Cielle Lockington, Celina Ruan, Gregg T. Tomy, James J. Petrik, Philippe J. Thomas, Alison C. Holloway

**Affiliations:** 1 Department of Obstetrics and Gynecology, McMaster University, Hamilton, ON L8S 4L8, Canada; peronog@mcmaster.ca (G.A.P.);; 2 Centre for Oil and Gas Research and Development, University of Manitoba, Winnipeg, MB R3T 2N2, Canadagregg.tomy@umanitoba.ca (G.T.T.); 3 Department of Biomedical Sciences, University of Guelph, Guelph, ON N1G 2W1, Canadajpetrik@uoguelph.ca (J.J.P.); 4 Wildlife and Landscape Science Directorate, Environment and Climate Change Canada, National Wildlife Research Center, Ottawa, ON L1A 0H3, Canada; philippe.thomas@ec.gc.ca

**Keywords:** polycyclic aromatic compounds, PAH, estrogen metabolism, 2-OHE2, 4-OHE2, DNA damage, apoptosis, granulosa cell, ovary

## Abstract

The expansion of the Alberta Oil Sands Region (AOSR) has increased the deposition of petroleum-derived chemicals into the surrounding environment. Among these, polycyclic aromatic compounds (PACs), including sulfur-containing heterocyclic hydrocarbons, have been detected in exposed local wildlife, yet the reproductive toxicity and genotoxicity of this suite of PACs remain largely unexplored. This study examined the effects of dibenzothiophene (DBT) and its alkylated congener, 2,4,7-trimethyldibenzothiophene (2,4,7-DBT), on estradiol (E2) synthesis and metabolism in granulosa cells (SIGCs). Cells were exposed to DBT or 2,4,7-DBT for 24 h at concentrations detected in AOSR wildlife tissues (0, 0.1, 1 and 10 nM). We measured the gene expression of markers involved in E2 synthesis, signaling and metabolism, E2 output via ELISA and E2 metabolite production via HPLC-MS/MS. Exposure to 2,4,7-DBT, but not DBT, shifted E2 metabolism towards 4-OHE2, a genotoxic E2 metabolite. DNA damage was assessed by γH2Ax expression, alongside DNA repair (*Parp1*) and survival markers (pAKT). Interestingly, both DBT and 2,4,7-DBT increased DNA damage and triggered apoptosis via a caspase-independent mechanism. Given the critical role of granulosa cells in steroidogenesis and fertility, these findings highlight the endocrine-disruptive effects of sulfur-containing heterocyclic PACs and their potential to compromise reproductive health in exposed mammals.

## 1. Introduction

The Alberta Oil Sands Region (AOSR) holds the third largest oil reserve in the world and spans three major deposits of recoverable bitumen: Athabasca, Peace River and Cold Lake [1]. Bitumen is a mixture of water, sand, clay and heavy crude oil that can be extracted via surface mining or in situ methods. With an estimated 160 billion barrels of recoverable bitumen, the Alberta oil sands are a vital contributor to both Alberta’s and Canada’s economies, producing over 3.3 million barrels of bitumen per day in 2023 [2]. As the global demand for petroleum and petroleum products continues to grow, oil extraction activities and production are projected to rise to 4 million barrels per day by 2033 [2].

In recent years, several reports have described the impact of these industrial activities on the surrounding environment and local wildlife [3]. Detection of contaminants related to oil extraction, such as polycyclic aromatic compounds (PACs), has increased in the atmosphere, lakes, sediments, rivers, snowpack and vegetation from areas near active oil extraction sites [4,5,6,7]. PACs are a diverse group of organic chemicals comprised of two or more conjugated hydrocarbon rings including polycyclic aromatic hydrocarbons (PAHs), heterocyclic PAHs, halogenated PAHs and alkylated PAHs [8]. The release of PACs into the surrounding environment can occur due to natural erosion of bitumen deposits or as a result of oil extraction, refining, upgrading, transport and storage [9]. Additionally, coal mining and the release of fugitive dust have contributed not only to elevated levels of petroleum-derived PACs in the local atmosphere but also their long-range transport and deposition into areas far from their emission sources [10,11,12]. Due to their widespread presence in the environment, wildlife living near industrialized areas such as the AOSR may be exposed to PACs, many of which are classified as carcinogens, mutagens and endocrine disruptors [7,13,14]. These contaminants may also contribute to reduced reproductive success in exposed biota [15,16]. In particular, priority PAHs have been extensively studied for their adverse effects on female reproductive health [13,17], including their role in premature ovarian insufficiency and infertility [18,19]. PAHs have also been shown to disrupt the synthesis of steroids, hormones and growth factors both in in vitro and in vivo models [20,21,22]. Collectively, the endocrine-disruptive effects of PAHs have been shown to weaken female fertility and limit reproductive success [21,23].

To date, there is limited knowledge regarding the impact of other classes of PACs like heterocyclic or alkylated PAHs despite their prevalence in the environment and biota [24,25,26]. Dibenzothiophene (DBT) is a heterocyclic sulfur-containing PAH commonly found in petrogenic sources and wastewaters associated with oil and gas extraction processes [27,28,29,30]. Studies in fish have assessed reproductive toxicity following DBT exposure. One study showed that 18-day exposure to DBT (0.271–1 μM) caused significant decreases in the hatching success of Japanese medaka (*Oryzias latipes*) [31], whereas another study showed 2-day exposure to DBT (0.05–10 μM) had no effect on the hatching success of zebrafish (*Danio rerio*) [32]. Alkylated PAHs, which are another class of PACs enriched in bitumen, have been reported to be five times more abundant than parent PAHs [29]. Recent studies have shown that alkylated congeners of DBT have been detected in higher concentrations in both petrogenic wastewaters and sediments as well as tissues of wildlife living near industrialized areas [26,33,34]. In a study assessing PAC and alkylated PAC load in fish (Northwest Atlantic cod), alkylated DBT was the major PAC detected in ovary and liver samples, which the authors attributed to nearby oil pollution [25]. Furthermore, in vitro assessment of these compounds in yeast and human adrenocortical carcinoma cells demonstrate that DBT and its alkylated congeners can alter the synthesis of hormones like estrogen and testosterone, posing a risk to reproductive functions in exposed animals [14,35,36]. Similarly, work from our group has previously shown that exposure to DBT, as well as its alkylated congener 2,4,7-trimethyldibenzothiophene (2,4,7-DBT), can impact estrogenic signaling and synthesis in placental trophoblast cells, with more pronounced effects observed from the alkylated congener [36].

With reports of the degree of alkylation impacting the estrogenic activity of the parent compounds [14], it is plausible that exposure to alkylated DBT congeners may also impair regulation of sex steroid synthesis and female reproductive success [37]. Given the critical role of the ovary in regulating female reproductive health, steroidogenesis and fertility, understanding the endocrine-disrupting effects of broader classes of PACs detected in exposed wildlife is essential. Therefore, this study aimed to determine the effects of DBT and its alkylated congener, 2,4,7-DBT, on estradiol (E2) synthesis and metabolism in granulosa cells, key cells for normal ovarian function. Furthermore, as other priority PAHs have been shown to be genotoxic [38], we further assessed DNA damage and apoptosis.

## 2. Results

### 2.1. Exposure to DBT and Alkylated DBT Alters Estrogen Signaling and Synthesis

**Figure 1:**
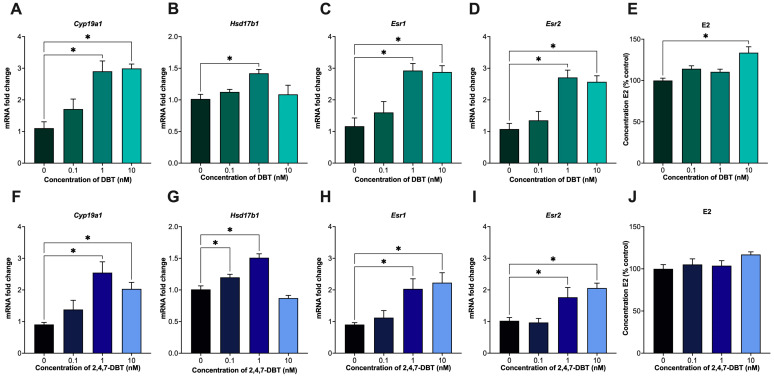
mRNA expression of enzymes involved in estrogen synthesis (*Cyp19a1*, *Hsd17b1*) and signaling (*Esr1*, *Esr2*) in SIGCs after 24 h treatment with 0 (vehicle, DMSO), 0.1, 1 and 10 nM dibenzothiophene (DBT) (**A**–**D**) and 2,4,7-trimethyldibenzothiophene (2,4,7-DBT) (**F**–**I**). Quantification of estradiol (E2) levels was measured via ELISA (**E**,**J**). Data are presented as mean ± SEM (*n* = 6) for gene data and as percent (%) control ± SEM (*n* = 6) for ELISA data. Statistical significance compared to control is denoted by asterisks: * *p* < 0.05.

In spontaneously immortalized rat granulosa cells (SIGCs), 24 h treatment with DBT and 2,4,7-DBT significantly increased the mRNA expression of key enzymes involved in estradiol synthesis and expression of estrogen receptors. *Cyp19a1* expression was significantly elevated by both compounds at 1 and 10 nM (Figure 1A,F). Additionally, DBT at 1 nM increased *Hsd17b1* expression (Figure 1B), while 2,4,7-DBT enhanced *Hsd17b1* at both 0.1 and 1 nM (Figure 1G). The effects on estradiol signaling were also assessed, where both DBT and 2,4,7-DBT increased the expression of estrogen receptors *Esr1* and *Esr2* at 1 and 10 nM (Figure 1C,D,H,I). Functionally, 10 nM DBT exposure increased E2 production, whereas 2,4,7-DBT did not have any significant effect on E2 output at any dose tested.

### 2.2. Exposure to 2,4,7-DBT Increases the Production of Estradiol Metabolite, 4-OHE2

**Figure 2:**
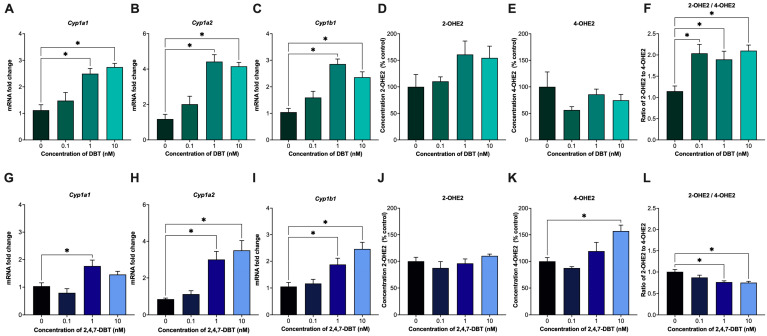
mRNA expression of cytochrome P450 (CYP) enzymes (*Cyp1a1*, *Cyp1a2*, *Cyp1b1*) involved in the metabolism of estradiol (E2) into catechol estrogens, 2-hydroxyestradiol (2-OHE2) and 4-hydroxyestradiol (4-OHE2) in SIGCs after 24 h treatment with 0 (vehicle, DMSO), 0.1, 1 and 10 nM dibenzothiophene (DBT) (**A**–**F**) or 2,4,7-trimethyldibenzothiophene (2,4,7-DBT) (**G**–**L**). Levels of 2-OHE2 and 4-OHE2 levels were measured via HPLC-MS/MS and presented as the ratio of 2-OHE2 to 4-OHE2 (**D**–**F**,**J**–**L**). Data are presented as mean ± SEM (*n* = 6) for gene data and as percent (%) control ± SEM (*n* = 6) for HPLC-MS/MS data. Statistical significance compared to control is denoted by asterisks: * *p* < 0.05.

Cytochrome P450 (CYP) enzymes play a major role in the metabolism of E2 into catechol estrogens. Specifically, CYP1A1 and CYP1A2 enzymes are largely responsible for converting E2 into 2-OHE2, while CYP1B1 predominantly converts E2 into 4-OHE2 [39]. In our study, treatment with 1 and 10 nM DBT or 2,4,7-DBT significantly increased the expression of *Cyp1a1* and *Cyp1a2* (Figure 2A,B,G,H) but had no significant effect on 2-OHE2 output for either chemical (Figure 2D,J). Interestingly, although both compounds increased *Cyp1b1* expression (Figure 2C,I), exposure to only 2,4,7-DBT significantly increased 4-OHE2 production at the 10 nM dose (Figure 2K).

The ratio of 2-OHE2 and 4-OHE2 has previously been measured in mammary tissues and the urine of patients with breast cancer, serving as a potential marker of hormone-induced oncogenesis [40,41]. A reduction in the 2-OHE2/4-OHE2 ratio was observed in patients with breast cancer compared to normal controls, demonstrating a dominance of 4-hydroxylation of E2 over 2-hydroxylation and preference of 4-OHE2 formation [40]. In this study, SIGCs exposed to DBT showed an increase in 2-OHE2/4-OHE2 at all doses tested (Figure 2F), whereas 2,4,7-DBT resulted in a decrease in this ratio at 1 and 10 nM (Figure 2L).

### 2.3. Both DBT and 2,4,7-DBT Cause DNA Damage in Granulosa Cells

**Figure 3:**
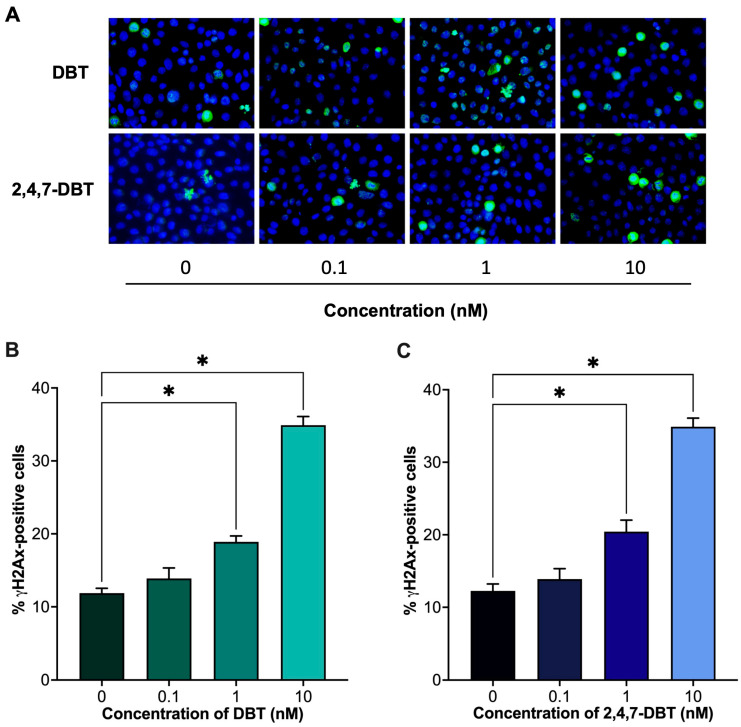
Immunofluorescence staining and quantification of γH2Ax-positive cells after 24 h treatment with 0 (vehicle, DMSO), 0.1, 1 and 10 nM dibenzothiophene (DBT) and 2,4,7-trimethyldibenzothiophene (2,4,7-DBT) in SIGCs. Representative immunofluorescent images of γH2Ax-positive foci (green) and nuclei stained with DAPI (blue) are shown (**A**). Quantification of the percentage of γH2Ax-positive cells after 24 h treatment with DBT (**B**). Quantification of the percentage of γH2Ax-positive cells after 24 h treatment with 2,4,7-DBT (**C**). Data are presented as mean ± SEM. Statistical significance compared to control is denoted by asterisks: * *p* < 0.05.

Some E2 metabolites have been previously shown to cause DNA damage [42]. As such, we further assessed whether exposure to DBT or 2,4,7-DBT altered the expression of γH2Ax, an established marker and surrogate for assessing DNA damage [43]. Immunofluorescence staining showed that both DBT and 2,4,7-DBT significantly increased the percentage of γH2Ax-positive cells in the 1 and 10 nM treatment groups (Figure 3).

### 2.4. DBT and 2,4,7-DBT Elicit Distinct Responses to DNA Damage, with 2,4,7-DBT Inducing Parp1 Expression and Activation of the AKT Signaling Pathway

**Figure 4:**
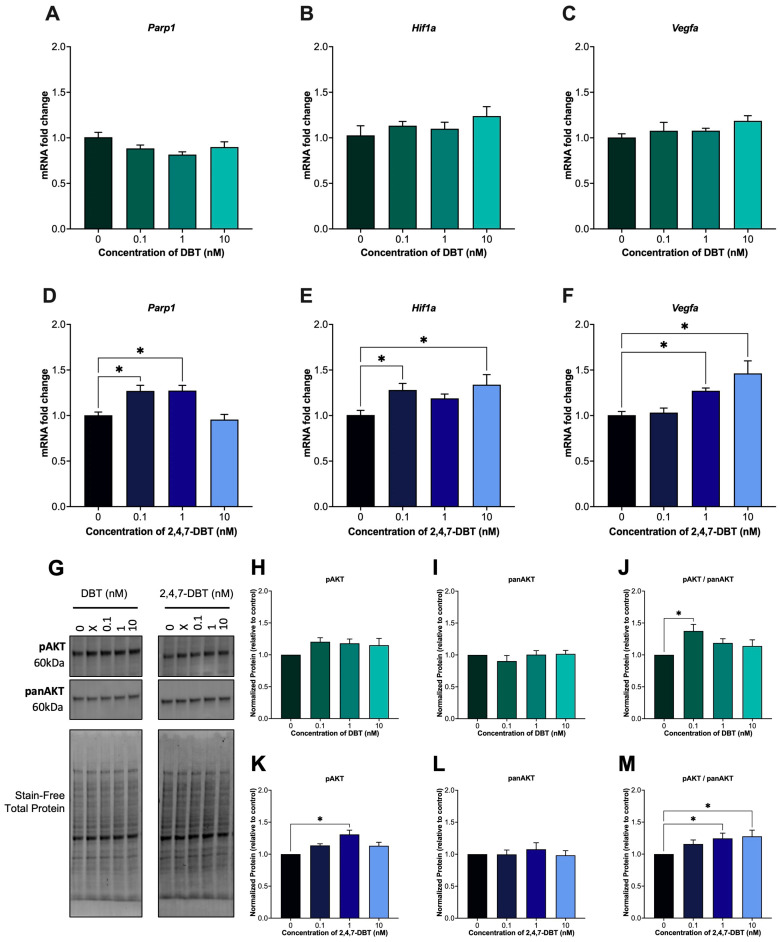
mRNA expression of poly(ADP-ribose) polymerase 1 (*Parp1*), hypoxia-inducible factor 1-alpha (*Hif1a*) and vascular endothelial growth factor A (*Vegfa*) and following 24 h treatment of SIGCs with 0 (vehicle, DMSO), 0.1, 1 and 10 nM dibenzothiophene (DBT) or 2,4,7-trimethyldibenzothiophene (2,4,7-DBT) (**A**–**F**). Protein levels of phosphorylated AKT (pAKT) and total AKT (panAKT) were assessed by Western Blot (**H**–**M**). Representative blots showing relative band density normalized to total protein of stain-free images (**G**). To account for blot-to-blot variability, fold changes were calculated by normalizing each sample to its corresponding control within the same blot. Data are presented as mean ± SEM (*n* = 6). Statistical significance compared to control is denoted by asterisks: * *p* < 0.05.

After 24 h exposure, 2,4,7-DBT significantly impacted DNA repair and the stress response pathways in SIGCs. The mRNA expression of *Parp1*, a key regulator of DNA damage and genomic stability, was significantly increased following treatment with 0.1 and 1 nM 2,4,7-DBT (Figure 4D) but remained unchanged with DBT exposure (Figure 4A). In addition to its role in DNA repair, PARP1 is known to also transcriptionally regulate oncogenes such as hypoxia-inducible factor 1 (*Hif1a*) and vascular endothelial growth factor A (*Vegfa*), both of which were upregulated following 2,4,7-DBT exposure only (Figure 4E,F). Interestingly, activation of protein kinase B (AKT), which can also regulate *Hif1a* and *Vegfa* expression, was increased by 0.1 nM DBT (Figure 4J) and by 1 and 10 nM 2,4,7-DBT (Figure 4M).

### 2.5. Exposure to 1 and 10 nM DBT or 2,4,7-DBT Increased Granulosa Cell Apoptosis via Caspase-Independent Mechanisms

**Figure 5:**
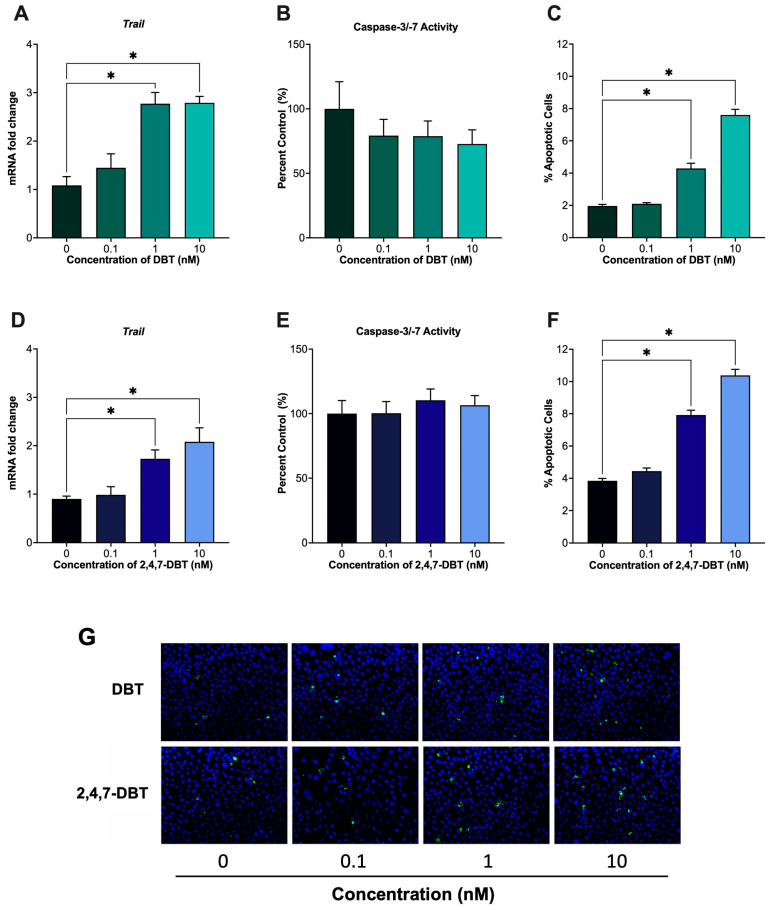
Assessment of apoptosis in SIGCs following 24 h treatment with 0 (vehicle, DMSO), 0.1, 1 and 10 nM dibenzothiophene (DBT) (**A**–**C**,**G**) and 2,4,7-trimethyldibenzothiophene (2,4,7-DBT) (**D**–**G**). Apoptosis was determined by mRNA expression of death receptor ligand, *Trail* ((**A**,**D**); *n* = 6) and caspase-3/-7 activity using a fluorescent cell-based assay ((**B**,**E**); *n* = 7–14) and via TUNEL staining ((**C**,**F**); *n* = 3). Representative images of TUNEL-positive cells (green) and nuclei stained with DAPI (blue) are shown (**G**). Data are presented as mean ± SEM. Statistical significance compared to control are denoted by asterisks * *p* < 0.05).

Despite their differential effects on DNA repair and stress response pathways, both DBT and 2,4,7-DBT increased DNA damage and apoptosis in SIGCs. Specifically, exposure to 1 and 10 nM of either chemical significantly increased the number of TUNEL-positive cells (Figure 5C,F), indicating increased apoptosis. Tumor necrosis factor-related apoptosis-inducing ligand (TRAIL) is a key pro-apoptotic protein that can mediate the extrinsic, death receptor-mediated apoptotic pathway, leading to a cascade of events that activate executioner caspases such as caspase 3 and caspase 7 [44]. Given the observed increases in TUNEL-positive cells, we assessed changes in the expression of *Trail* and caspase-3/-7 activity. At the same concentrations that increased TUNEL-positive cells, exposure to DBT and 2,4,7-DBT upregulated *Trail* mRNA expression (Figure 5A,D), suggesting activation of death receptor signaling pathways. However, downstream caspase-3/-7 activity remained unchanged (Figure 5B,E). Therefore, although extracellular apoptotic pathways may be induced, cell death likely occurs through caspase-independent mechanisms downstream of TRAIL.

## 3. Discussion

While the genotoxic and reproductive effects of prototypical PAHs such as benzo(a)pyrene are well established [45], less is known about the broader classes of PACs, including DBT and its alkylated congeners, despite their environmental prevalence and detection in exposed biota [7,25,46]. Notably, environmental concentrations of DBTs have been reported at levels up to 57-fold higher than in pre-oil sands development baselines, with alkylated congeners comprising a greater proportion of total DBT burdens in exposed animals [26,47]. Given evidence from animal and in vitro models showing that priority PAH exposure impairs follicle development and induces apoptosis and DNA damage [18,23,48,49,50], understanding how DBT and alkylated DBT exposure may similarly disrupt ovarian function is needed to assess potential impacts on mammalian reproductive health.

In this study, DBT and 2,4,7-DBT significantly altered estrogen synthesis and metabolism in granulosa cells. Both compounds increased the expression of estrogenic enzymes (*Cyp19a1*, *Hsd17b1*), but only DBT significantly increased E2 levels (Figure 1). The increase in E2 output is consistent with observations made in human H295R adrenocortical carcinoma cells exposed to DBT, where the authors also reported increases in E2 levels following exposure to C1-C3 alkylated DBT congeners [14]. Similarly, previous work from our group found that only 2,4,7-DBT significantly increased E2 production in human HTR-8/SVneo placental trophoblasts following 48 h exposure [36]. Such discrepancies in E2 synthesis may reflect tissue- and cell-specific differences in enzyme or receptor expression involved in E2 synthesis and metabolism across species [51,52].

E2 is metabolized into 2-OHE2 and 4-OHE2 by CYP1A1/2 and CYP1B1, respectively [39]. We observed that both compounds increased mRNA expression of E2 metabolizing enzymes (*Cyp1a1*, *Cyp1a2*, *Cyp1b1*); however, only 2,4,7-DBT altered individual E2 metabolite levels (Figure 2). The induction of CYP enzymes is typically mediated via activation of the aryl hydrocarbon receptor (AhR) [53]. Previous studies in rat hepatoma (H4IIE), human hepatoma (AZ-AhR) and fish liver (RTL-W1) cell lines reported that DBT induced weak-to-no AhR activity [54,55] and failed to induce CYP1A activity in EROD assays [54]. As such, the increases in the CYP enzyme expressions observed in this study were unexpected. Interestingly, Lam et al. (2018) showed that while DBT alone did not induce AhR activity after 24 h, alkylation of the parent compound significantly enhanced AhR activation [56]. Specifically, 2-methyldibenzothiophene and 2,8-dimethyldibenzothiophene elicited 24% and over 80% of the maximal TCDD induction, respectively [56]. Therefore, the increased expression of CYP enzymes and elevated metabolism of E2 into 4-OHE2 observed with 2,4,7-DBT in our study may reflect enhanced AhR activation in granulosa cells, potentially driven by its higher degree of alkylation (Figure 2).

4-OHE2 is a catechol estrogen with known genotoxic potential, whereas 2-OHE2 is thought to be protective [39]. An accumulation of 4-OHE2 leads to the formation of reactive quinones that can form depurinating DNA adducts [57]. We observed that SIGCs exposed to 1 and 10 nM 2,4,7-DBT had a reduced ratio of 2-OHE2/4-OHE2, indicating a shift towards 4-OHE2 dominance, which corresponded with increased DNA damage at the same doses (Figure 3). This is consistent with previous findings in normal breast epithelial cells (MCF-10A), where 4-OHE2 treatment induced DNA damage and apoptosis, effects that were reversed by antioxidant treatment [58]. Similarly, in human breast cancer cells (MCF-7), 4-OHE2 treatment and inhibition of catechol-O-methyltransferase (COMT), the enzyme responsible for the breakdown of catechol estrogens into methoxyestradiol, induced γH2Ax expression in response to DNA damage [59]. Although DBT exposure did not alter 2-OHE2 or 4-OHE2 levels, we still observed a significant increase in DNA damage (Figure 3). To our knowledge, only one study conducted in HepG2 cells has shown DNA adduct formation following 48 h treatment with DBT (50 and 150 μM) [60]. The DNA damage observed in our study may instead be linked to increased E2 levels, as evidence in breast cancer cells has shown that E2 can induce γH2Ax expression and DNA double-stranded breaks [61], an effect that could be mediated via E2-ERα activity [38,61].

In response to DNA damage, cells activate a DNA damage response (DDR) that detects DNA breaks and initiates DNA repair signaling pathways [38]. Poly(ADP-ribose) polymerase-1 (PARP1) is a key DDR protein that is involved in DNA repair, transcriptional regulation, stress responses and cell death [62]. Overexpression of PARP1 has been reported in several malignancies, including breast, uterine and ovarian cancers, where it contributes to cell survival, mutagenesis and tumor progression [63]. Notably, PARP1 expression has also been altered by 4-OHE2 treatment in normal breast epithelial cells (MCF-10A), linking estrogen metabolism to DDR activation [64]. In our study, we see a matched increase in *Parp1* mRNA expression with 4-OHE2 and DNA damage at the 1 nM 2,4,7-DBT dose (Figure 4). To further assess downstream PARP1 signaling, we measured the expression of hypoxia-inducible factor 1 (*Hif1a*) and vascular endothelial growth factor alpha (*Vegfa*), both of which are involved in hypoxic and pro-angiogenic responses and are often dysregulated in cancer [63,65]. *Hif1a* was significantly upregulated at 0.1 and 10 nM, while *Vegf* increased at 1 and 10 nM with 2,4,7-DBT exposure (Figure 4). Interestingly, prior studies have also shown that 4-OHE2, but not 2-OHE2, can increase *Hif1a* and *Vegf* via the PI3K/AKT/FRAP pathway in ovarian cancer cells (OVCAR-3) [66]. Similarly, we observed increased phosphorylation of protein kinase B (pAKT) at 1 and 10 nM 2,4,7-DBT, which may contribute to *Hif1a* and *Vegfa* upregulation at these doses. Together, these findings suggest that 2,4,7-DBT, but not DBT, causes 4-OHE2 accumulation, which initiates DDR and induces signaling pathways involving PARP1 and AKT activation. While these markers are often linked to survival and angiogenesis in cancer, the results from our study may instead reflect a stress response to DNA damage in our model.

To determine whether the observed DNA damage and DDR impacted cell fate, we assessed whether DBT or 2,4,7-DBT exposure led to apoptosis. Both compounds significantly increased the percentage of TUNEL-positive cells at the same doses, which induced DNA damage (Figure 5C,F), suggesting that the DDR mechanisms induced by 2,4,7-DBT were not sufficient to promote cell survival. Interestingly, while we observed induction of death receptor pathways like *Trail*, caspase-3/-7 activity remained unchanged (Figure 5A,B,D,E), suggesting that apoptosis occurred via caspase-independent mechanisms for both compounds. Previous studies have shown that overactivation of PARP1 can trigger caspase-independent cell death, which is characterized by mitochondrial disruption and nuclear DNA fragmentation [67]. In SK-N-SH neuroblastoma cells, DBT has been shown to cause significant alterations in mitochondrial membrane potential, promoting apoptosis [68]. While an increase in E2 and subsequent apoptosis was unexpected following DBT treatment, it has been reported that mitochondria can remain intact in cells undergoing apoptosis (condensed or fragmented DNA) until total cell collapse [69,70]. This phenomenon is hypothesized to result from cytoskeletal reorganization and clustering of intracellular organelles, which could facilitate coupling between mitochondria and lipid droplets to enable steroidogenesis [69,70].

## 4. Materials and Methods

### 4.1. Cell Culture Maintenance and Treatment

Spontaneously immortalized granulosa cells (SIGCs, passages 12–17) were maintained in 100 mm dishes (Corning) in DMEM/Ham’s F-12 media with L-glutamine (DMEM/F12, Corning, Corning, NY, USA), supplemented with 10% (*v*/*v*) fetal bovine serum (HyClone^TM^, Marlborough, MA, USA) and 2% (*v*/*v*) penicillin–streptomycin (Gibco, Grand Island, NY, USA) in a humidified atmosphere of 95% O_2_ and 5% CO_2_ at 37 °C.

To assess changes in mRNA expression, SIGCs were seeded at 250,000 cells/well in six-well tissue culture plates (Falcon^®^, Corning, NY, USA) and were allowed to adhere for 24 h. Cells were subsequently exposed to 0 (vehicle, DMSO), 0.1, 1 and 10 nM DBT or 2,4,7-DBT (Toronto Research Chemicals, North York, ON, Canada) (*n* = 6 independent experiments) for an additional 24 h, after which cells were harvested for subsequent analyses (described below). To date, most studies assessing DBT toxicity have assessed high concentrations of DBT (ranging from 0.05–100 μM), far exceeding those typically detected in environmental tissues of exposed wildlife and biota. Therefore, in our study, our selected dose range is representative of levels detected in tissues of wildlife exposed to accidental crude oil spills [71] and collected near active bitumen extraction sites in the Alberta oil sands region [72,73].

### 4.2. RNA Isolation and Quantitative Real-Time PCR

Following 24 h treatment, RNA was extracted from cells using TRIzol^®^ Reagent (Invitrogen, Waltham, MA, USA), and total RNA concentrations were calculated using the NanoDrop^TM^ One Microvolume UV-Vis Spectrophotometer (Thermo Scientific^TM^, Waltham, MA, USA). Complementary DNA (cDNA) was synthesized from 2 ug of total RNA using the High-Capacity cDNA Reverse Transcription Kit (Applied Biosystems, Waltham, MA, USA).

Quantitative real-time PCR (qPCR) was performed to measure the mRNA expression of key genes involved in estradiol (E2) synthesis, signaling and metabolism, as well as the DNA damage response, using PerfeCTa SYBR^®^ Green FastMix (Quantabio, Beverly, MA, USA) on the CFX384 Touch^TM^ Real-Time PCR Detection System (Bio-Rad Laboratories, Hercules, CA, USA). The genes analyzed included aromatase (*Cyp19a1*) and hydroxysteroid 17-beta dehydrogenase 1 (*Hsd17b1*), which are critical enzymes for E2 synthesis; estrogen receptor alpha (*Esr1*) and estrogen receptor beta (*Esr2*), key mediators of estradiol signaling; cytochrome P450 1A1 and 1A2 (*Cyp1a1*, *Cyp1a2*), enzymes responsible for converting E2 into 2-hydroxyestradiol (2-OHE2); and cytochrome P450 1B1 (*Cyp1b1*), an enzyme responsible for converting E2 into 4-hydroxyestradiol (4-OHE2). We also assessed expression of poly(ADP-ribosyl) polymerase 1 (*Parp1*), a key marker involved in DNA repair, and its downstream markers, hypoxia-inducible factor 1-alpha (*Hif1a*) and vascular endothelial growth factor alpha (*Vegfa*), and tumor necrosis factor-related apoptosis-inducing ligand (*Trail*), which is involved in initiating apoptosis through extracellular activation of death receptors. Relative gene expressions were calculated using the ΔΔCt method [74]. Briefly, Ct values for each gene of interest were first normalized to the geometric means of two validated reference genes (β2 microglobulin (*B2mg*) and hypoxanthine phosphoribosyl transferase 1 (*Hprt*)), which were confirmed to be stable following exposure to DBT and 2,4,7-DBT. These normalized values were then compared to the average of the control group and transformed using 2^−ΔΔCt^ to determine the relative mRNA fold change. Primer sequences are provided in Table 1.

### 4.3. Protein Isolation and Western Blot

SIGCs grown in 100 mm dishes were exposed to 0 (vehicle, DMSO), 0.1, 1 or 10 nM DBT or 2,4,7-DBT (*n* = 6 independent experiments) for 24 h. Following treatment, spent media were collected for hormone measurement (described below), and total protein was collected and homogenized in lysis buffer (50 mM HEPES, 150 mM NaCl, 10 mM NaF, 10 mM sodium pyrophosphate, 5 mM EDTA, 250 mM sucrose, 1 mM DTT, 1 mM sodium orthovanadate, 1% Triton X-100 and one tablet of cOmplete^TM^ Protease Inhibitor Cocktail (Sigma-Aldrich, St. Louis, MI, USA)). Quantification of total protein was determined using a Pierce BCA Protein Assay Kit (Thermo Scientific^TM^) following the manufacturer’s protocol. Protein was then standardized and denatured at 95 °C for 5 min.

Protein samples (20 μg) were separated by gel electrophoresis using 10% Mini-PROTEAN TGX Stain-Free Protein Gels (Bio-Rad Laboratories). Protein was transferred onto a low fluorescence PVDF membrane using the Trans-Blot Turbo Transfer apparatus (Bio-Rad Laboratories) and then imaged for total protein using the ChemiDoc MP Imaging System (Bio-Rad Laboratories). Membranes were blocked with 5% BSA or fat-free milk made in Tris-buffered saline with 0.1% Tween^®^20 (TBST) for 20 h at 4 °C and then incubated with rabbit monoclonal anti-pAKT (Cell Signaling Technology, Danvers, MA, USA; 4058, 1:1000) or rabbit monoclonal anti-panAKT (Cell Signaling Technology, 4685, 1:1000) overnight at 4 °C. After three washes with TBST, membranes were incubated with HRP-conjugated goat anti-rabbit secondary antibody (1:10,000, Abcam, ab7090, Cambridge, UK) for 1 h, followed by another three washes with TBST. Proteins were detected using the Clarity Max Western ECL Substrate (Bio-Rad Laboratories) and captured using the ChemiDoc MP Imaging System. Images were analyzed using Image Lab software (BioRad, V6.1.0) and normalized to total protein [75,76]. To account for blot-to-blot variability, fold changes were calculated by normalizing each sample to its corresponding control within the same blot.

### 4.4. Estradiol Quantification via ELISA

Spent media collected from SIGCs treated for 24 h with DBT or 2,4,7-DBT were used to assess the production of 17-β-estradiol using a commercially available kit according to the manufacturer’s protocol (17-β-Estradiol ELISA Kit, Enzo Life Sciences, Farmingdale, NY, USA). Absorbance was measured using the Synergy^TM^ H1 microplate reader (Aligent Technologies, Santa Clara, CA, USA) and fit to a four-parameter logistic regression curve to determine concentrations of 17-β-estradiol using the free online GainData^®^ ELISA calculator (arigo Biolaboratories Corp., Zhubei City, Taiwan).

### 4.5. Hydroxyestradiol Quantification via HPLC-MS/MS

To assess changes in hydroxyestradiol production, we performed HPLC-MS/MS using the following reagents: mass-labeled standard of 2-OHE2 (^13^C_6_-2-OHE2; Cambridge Isotope Laboratories; Tewksbury, MA, USA); unlabeled analytical standards of 2-OHE2 and 4-OHE2 (Cayman Chemical Company; Ann Arbor, MI, USA); and HPLC-grade methanol, Optima^TM^ LC/MS-grade water and formic acid (all from Fisher Scientific; Ottawa, ON, Canada).

A 500 µL aliquot of spent media was diluted in 490 µL of methanol, spiked with 10 µL of 5 ng/µL ^13^C_6_-2OHE2 internal standard and vortexed for 30 s. This mixture was transferred to an LC vial, and 15 µL was injected onto an Agilent Infinity Lab Poroshell 120 HPH-C18 HPLC column (2.1 × 50 mm, 2.7-micron) using a CTC Pal autosampler. An Agilent 1100 LC binary pump (G1312A) was used with the following binary gradient to separate 2- and 4-OHE2: water with 0.1% formic acid (solvent A) and methanol (solvent B)—0–3.00 min hold at 10% B, 3.01–12.00 min linear ramp to 90% B, 12.01–13.00 min hold at 90% B, 13.01–14.00 min at 10% B and 14.01–17.00 min hold at 10% B. The flow rate was 200 µL/min, and the column temperature was held at 42 °C. Mass analysis was performed in negative mode electrospray ionization via a triple quadrupole mass spectrometer (SCIEX API 365) (AB Sciex LLC; Marlborough, MA, USA) with an HSID ionics EP 10+ orthogonal ionization source. The ionspray voltage was maintained at 4500 V, and the capillary was held at 250 °C. The pseudo ion transitions for quantitation were 2-OHE2: *m*/*z* 287.2 → *m*/*z* 287.2; 4-OHE2: *m*/*z* 287.2 → *m*/*z* 287.2 and 13C6-2OHE2: *m*/*z* 293.1 → *m*/*z* 293.1. Quantitation of 2- and 4-OHE2 in samples was based on isotope dilution using 13C6-2OHE2 as the internal standard.

### 4.6. Immunofluorescence Microscopy for γH2Ax

Histone H2Ax is a vital protein involved in nucleosome assembly, chromatin remodeling and DNA repair. Upon DNA damage, H2Ax is phosphorylated at the Ser139 residue, resulting in the formation of γH2Ax [77]. To assess DNA damage due to DBT or 2,4,7-DBT exposure, SIGCs were seeded in eight-well chamber slides (Falcon^®^) at 30,000 cells/well and were treated as described previously (*n* = 3). Cells were fixed with 10% neutral-buffered formalin (Sigma) for 1 h at room temperature, washed with Dulbecco’s PBS and permeabilized using 0.1% Triton X-100 (Fisher Scientific) for 15 min. Cells were blocked with 5% BSA for 1 h and were incubated with rabbit anti-yH2Ax (abcamAb2893; 1:100) overnight at 4 °C. Alexa Fluor 488-conjugated secondary antibody (Invitrogen A21206; 1:400) was applied to the samples for 1 h at room temperature. After washing, samples were counterstained using ProLong^TM^ Gold Antifade Mountant with DAPI (Invitrogen) and mounted with coverslips. Images were obtained using an Olympus microscope, and image analyses were conducted using Image J. Immuno-positive nuclei were counted and expressed as a percentage of total cells.

### 4.7. Caspase-3/-7 Activity

Caspase-dependent apoptosis was assessed using the CellEvent^TM^ Caspase-3/-7 Green Detection Reagent kit (Invitrogen), according to the manufacturer’s instructions. Briefly, SIGCs were seeded in a 96-well plate at 12,000 cells/well, allowed to adhere and then treated with DBT or 2,4,7-DBT as described above (*n* = 7–14 per treatment group). Following 24 h treatment, detection reagent was added to each well and then incubated for 30 min at 37 °C in a humidified atmosphere. Fluorescence intensity was measured at 502/530 nm (excitation/emission) using the Synergy^TM^ H1 microplate reader.

### 4.8. TUNEL Assay

Apoptosis was determined via TUNEL (terminal deoxynucleotidyl transferase dUTP nick-end labeling) staining using the In Situ Cell Death Detection Kit, Fluorescein (Roche, Basil, Switzerland). Briefly, SIGCs were seeded and exposed to DBT or 2,4,7-DBT in eight-well chamber slides as described above. Following fixation with 10% neutral-buffered formalin for 1 h at room temperature, the SIGCs were washed twice and permeabilized with 0.1% Triton X-100 in 0.1% sodium citrate. RQ1 RNase-Free DNAse (Promega Corp., Madison, WI, USA) made in 50 mM Tris-HCl was used as a positive control. Samples were then incubated with the fluorescein-labeled TUNEL enzyme for 1 h at 37 °C in a humidified atmosphere. Samples were counterstained and mounted using the ProLong^TM^ Gold Antifade Mountant with DAPI and imaged on an Olympus BX-61 microscope (Olympus, Japan). The percentage of TUNEL-positive nuclei was determined by taking the average of the total number of nuclei present in five fields of view per slide per treatment group.

### 4.9. Statistical Analysis

All data were analyzed for statistical significance using GraphPad Prism (v10.4.1, Boston, MA, USA). Outcome measures were assessed for outliers using Grubb’s outlier test and tested for normality (Shapiro–Wilk test) and equal variance (Brown–Forsythe test). Comparisons between control and treatment groups were conducted using one-way ANOVA, with pairwise comparisons using the Dunnett method. For data that did not meet the assumptions of normality or equal variance, a Kruskal–Wallis one-way ANOVA non-parametric test was used, followed by a Dunn’s post-hoc test for comparisons against control. Data were presented as mean ± SEM and were considered significant when *p* < 0.05.

## 5. Conclusions

Overall, these findings demonstrate that while DBT and 2,4,7-DBT differentially alter estrogen synthesis and metabolism, exposure to either compound leads to DNA damage and apoptosis of key cells within the ovary. Few studies have investigated the reproductive impacts of PACs associated with oil extraction activities in mammals, and even fewer have assessed endocrine-disruptive effects. To our knowledge, no study has assessed the effects on E2 metabolism. Given that several PACs are known to induce CYP activity, enzymes that are responsible for metabolizing E2, our study highlights the importance of assessing E2 metabolism and hydroxyestradiol formation to better understand the endocrine-disruptive effects of PACs in exposed wildlife. In addition to measuring downstream E2 metabolites, future studies should incorporate protein assessments of key metabolizing enzymes (i.e., Western Blot or enzyme activity assays) to further strengthen our mechanistic understanding into how sulfur-containing PACs disrupt estrogen dynamics. Furthermore, while petrogenic sources remain a large contributor to PACs and DBTs, the widespread detection of these chemicals in PM2.5 air samplers and vehicle exhaust from urban and high-traffic areas highlights the broader environmental and toxicological relevance for this class of contaminants [78,79,80]. This study focused on individual heterocyclic PAHs and provides a novel mechanism by which sulfur-containing and alkylated PAHs may disrupt ovarian function. Future work should investigate whether alterations in E2 metabolism and ovarian apoptosis occur following exposure to complex mixtures and assess the broader implications for female fertility and reproductive health in wildlife living in close proximity to oil extraction sites.

## Figures and Tables

**Table 1:** Primer sequences for qPCR.

Accession Number	Gene Name	Symbol	Forward Sequence (5′–3′)	Reverse Sequence (5′–3′)
NM_012512.2	Beta-2 microglobulin	B2m	AATTCACACCCACCGAGACC	GCTCCTTCAGAGTGACGTGT
NM_012583.2	Hypoxanthine phosphoribosyl transferase 1	Hprt	GCAGTACAGCCCCAAAATGG	GGTCCTTTTCACCAGCAAGCT
NM_012851.2	Hydroxysteroid 17-Beta Dehydrogenase 1	Hsd17b1	AAGGTCTGTGCGAGAGTCTG	TTTCATGGAAGGCTGTGTGC
NM_017085.2	Cytochrome P450 Family 19 Subfamily A Member 1	Cyp19a1	CTGTCCA TTCCAGCACCC TT	AGTAGTTTGGCTGTGGCTC C
NM_012689.1	Estrogen Receptor 1	Esr1	GCTCCACTTCAGCACATTCC	GGATTCGCAGAACCTTGTGG
NM_012754.3	Estrogen Receptor 2	Esr2	TGAGGCGGACAGACTACAGA	GGTAAGGGGTGCGTAACCAA
NM_012540.3	Cytochrome P450 Family 1 Subfamily A Member 1	Cyp1a1	TTGGGGAGGTTACTGGTTCTG	GAGTTAGGGAGGTAACGGAGG
NM_012541.3	Cytochrome P450 Family 1 Subfamily A Member 2	Cyp1a2	AGGGAATGCTGTGGACTTCTT	AGTGTTCCTGGACTGTTTTCTG
NM_012940.2	Cytochrome P450 Family 1 Subfamily B Member 1	Cyp1b1	CAACCCAACTTACCATACGTCA	AAACAAAGGTGTTGGCAG
NM_013063.2	Poly(ADP-Ribose) Polymerase 1	Parp1	AAGTGCGAACTACTGCCACA	CTTTGACACTGTGCTTGCCC
NM_024359.1	Hypoxia-Inducible Factor 1 Subunit Alpha	Hif1a	CAGACCCAGTTACAGAAACCTAC	GATTCATCAGTGGTGGCAGTTG
NM_031836.3	Vascular Endothelial Growth Factor A	Vegfa	TCTCCCAGATCGGTGACAGT	AAGGAATGTGTGGTGGGGAC
NM_145681.2	Tumor Necrosis Factor-Related Apoptosis-Inducing Ligand	Trail	GGACGGAGGGAGGATCAAAC	CAGTATGGGATCGGGGTAGC

## Data Availability

The original contributions presented in this study are included in the article. Further inquiries can be directed to the corresponding author.

## Abbreviations

The following abbreviations are used in this manuscript:

AOSR	Alberta oil sands region
PACs	Polycyclic aromatic compounds
PAHs	Polycyclic aromatic hydrocarbons
DBT	Dibenzothiophene
2,4,7-DBT	2,4,7-Trimethyldibenzothiophene
SIGCs	Spontaneously immortalized granulosa cell
TUNEL	Terminal deoxynucleotidyl transferase dUTP nick-end labeling
AhR	Aryl hydrocarbon receptor
CYP	Cytochrome P450
EROD	Ethoxyresorufin-O-deethylase
TCDD	2,3,7,8-tetrachlorodibenzo-p-dioxin
COMT	Catechol-O-methyltransferase
DDR	DNA damage response
PARP1	Poly(ADP-ribose) polymerase-1
AKT	Protein kinase B
pAKT	Phosphorylated protein kinase B
